# Regulation of bone health through physical exercise: Mechanisms and types

**DOI:** 10.3389/fendo.2022.1029475

**Published:** 2022-12-07

**Authors:** Xinyu Chang, Sheng Xu, Hao Zhang

**Affiliations:** ^1^ Department of Traumatic Orthopedics, Changhai Hospital, Naval Medical University, Shanghai, China; ^2^ National Key Laboratory of Medical Immunology and Institute of Immunology, Institute of Immunology, Naval Medical University, Shanghai, China; ^3^ Department of Traumatic Orthopedics, the First Affiliated Hospital of Naval Medical University, Naval Medical University, Shanghai, China

**Keywords:** osteoporosis, bone mass, exercise, cytokines, irisin, Piezo1, PCs

## Abstract

Osteoporosis, characterized by bone mineral density reduction, bone mass loss, increased bone fragility, and propensity to fractures, is a common disease in older individuals and one of the most serious health problems worldwide. The imbalance between osteoblasts and osteoclasts results in the predominance of bone resorption and decreased bone formation. In recent years, it has been found that regular and proper exercise not only helps prevent the occurrence of osteoporosis but also adds benefits to osteoporosis therapy; accordingly, bone homeostasis is closely associated with mechanical stress and the intricate crosstalk between osteoblasts and osteoclasts. In this review, we summarize the mechanisms of exercise on osteoporosis and provide new proposals for the prevention and treatment of osteoporosis.

## 1 Introduction

Osteoporosis is a metabolic bone disease characterized by the loss of bone mineral density and the deterioration of bone quality *via* microarchitectural and biomechanical alterations. The bone mass reduction in osteoporosis results in skeletal fragility and increases the fracture risk. Approximately 1.5 million new fractures are reported worldwide each year, resulting in a huge economic burden for the affected families and countries (1).

The cellular and molecular mechanisms underlying osteoporosis are multifactorial, reflecting the complex interactions of the diverse cells in the bone marrow microenvironment. Osteoporosis is considered to be a direct result of the reduction of bone mass and imbalance in bone remodeling. Human bone mass is determined by the intricate bone microenvironment led by the dynamic balance of osteoblasts, osteoclasts, and other cells. Bone remodeling imbalance is responsible for several pathological conditions, including osteosclerosis, osteopetrosis, and osteoporosis. The imbalance within the bone microenvironment is not only closely related to menopause and aging, but is also associated with the occurrence and development of inflammation.

Depending on its primary cause, osteoporosis is divided to primary osteoporosis and secondary osteoporosis. Primary osteoporosis includes postmenopausal osteoporosis, senile osteoporosis, and juvenile osteoporosis, while secondary osteoporosis occurs due to drugs, such as long-term glucocorticoid administration in large doses, long-term limb disuse, or in other diseases.

Current solutions to reduce the incidence of osteoporosis include identifying persons at risk, prescribing long-term pharmaceutical treatment, and reducing the risk of falls. In addition, its prevention strategies include sufficient calcium and vitamin D intake, cessation of tobacco or alcohol consumption, and being physically active at young age to increase and maintain peak bone mass (2). However, many researchers have recently found that exercise is beneficial for preventing and treating osteoporosis (3–5).

## 2 Bone remodeling and bone microenvironment

Osteoblasts, osteoclasts, and osteocytes are the fundamental cell types in the bone microenvironment, which interact and cooperate with each other on the basis of extracellular communication to coordinate bone remodeling (6). The precursors of osteoblasts originate from bone marrow mesenchymal stem cells (BMSCs) (7, 8). In the period of bone formation, osteoblast precursors on the bone surface generate calcified matrix, also called mineralized matrix. When osteoclast precursors, which differentiate from bone marrow hematopoietic stem cells, contact with the mineralized matrix, they polarize and fuse to multinucleated giant cells, giving rise to osteoclasts. Osteoclasts form specific domains, including the ruffled border, the sealing zone, and the functional secretory domain, which are important for the function of bone resorption reabsorption (9, 10). Bone resorption is the precondition for the formation of resorption lacuna. Osteoblasts express and secrete two necessary cytokines, including macrophage colony-stimulating factor M-CSF and the ligand for the receptor activator of nuclear factor kB (RANKL). Under the stimulation by the two cytokines, hematopoietic cells differentiate to osteoclasts. Likewise, osteoclasts generate MMP-2, 4, 6, and 7, which are important for recruiting osteoblasts and their subsequent activation (11). Osteocytes differentiate from osteoblasts. Mature osteocytes are rooted in the bone matrix and are connected to each other by stretched dendritic cell extensions through a network formed by canaliculi, called lacunocanalicular network (LCN).

## 3 Mechanisms of exercise on bone health

Mechanical strain, such as during exercise, is an important physiological factor that regulates bone formation in living organisms (12). The mechanism is based on the function of osteocytes, which perceive, transform, and transmit mechanical loading. Osteocytes are the main bone cells capable of transforming mechanical stimuli into intercellular signals through the exceedingly specialized LCN. In the state of exercise, osteocytes are able to sense variation in mechanical stress, respond and send signals to the surrounding cells, and regulate functions of osteoblasts, osteoclasts, and extracellular matrix (ECM) of the bone microenvironment (13). Transduction of the mechanical signals to other cells in the microenvironment leads to the upregulation of bone turnover and subsequent bone deposition (14). Other cells, such as osteoblasts, can also sense mechanical stimuli (15, 16).

Osteocytes are able to respond to physical stimulation directly by making use of various mechanosensors, such as Wnt signaling components (17), integrins sensing extracellular matrix stress (18, 19), connexins forming intercellular junctions (20, 21), and purinergic receptors (22). The function of osteocytes in response to mechanical stimulation from exercise is also regulated indirectly by extracellular cytokines ranging from RANKL/OPG to sclerostin (23).

**Figure 1:**
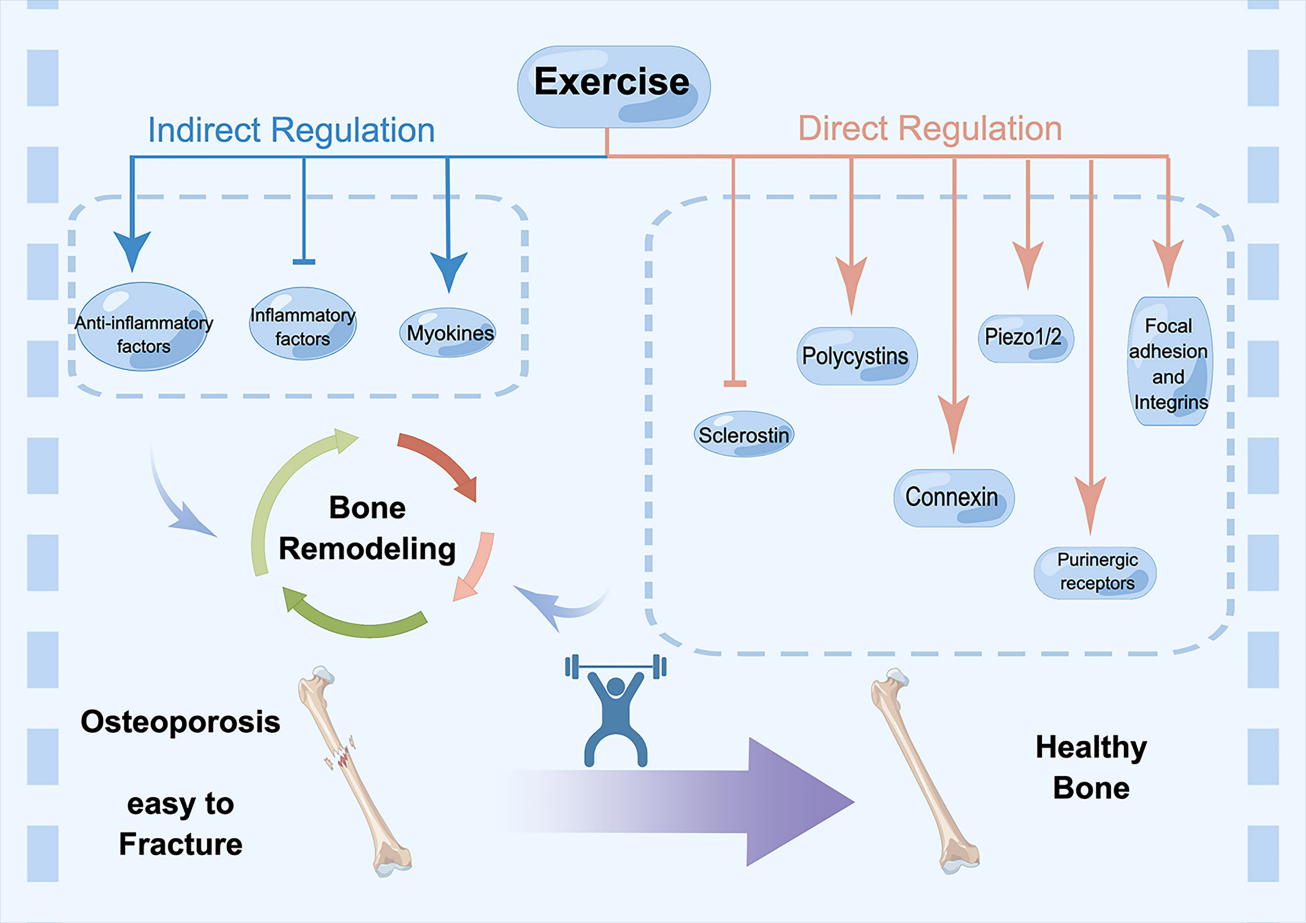
Direct and indirect regulation mechanisms of exercise for osteoporosis. Cell surface receptors are the starting point for understanding the regulatory effects of mechanical loading, which include focal adhesions, integrins, purinergic receptors, connexin, polycystins, Piezos, Wnt signaling and sclerostin. In addition, exercise-induced regulation of bone environment also occurs through cytokines, including inflammatory factors and myokines, such as irisin. These factors affect bone remodeling collectively. Exercise promotes bone mass and sustain healthy bone, while lacking exercise results in osteoporosis and fracture.

Here, we gathered and curated information related to mechanical signaling mechanisms (**Figure 1**).

### 3.1 Direct regulation of cell surface receptors

Cell surface receptors are vital signal‐sensing molecules that respond to extracellular environmental changes. This phenomenon is the starting point for understanding the regulatory effects of mechanical loading. Many proteins on the cell surface have been reported to act as mechanical transducers in bone cells, which include focal adhesions (24), integrins (25), purinergic receptors (22), connexin (20, 21), polycystins (26, 27), Piezos (28, 29), Wnt signaling and sclerostin.

#### 3.1.1 Focal adhesions and integrins

Focal adhesion complex is the most important component of integrin-based adhesion complex, which transduces mechanical stress from ECM into cytoplasm (18). Integrin proteins include α and β subunits, where integrin α senses different signals out of the cells, while integrin β delivers signal pathways in the cells (19). The integrin family consists of 24 members, which were discovered in the past two decades. Different tissues express different types of integrin, and proteins in the ECM help determine which type of integrin these tissues express. Integrins are expressed in various bone cells, such as osteoblasts, osteocytes, and osteoclasts.

During exercise, focal adhesion enables osteocytes to sense mechanical forces. With the help from other different adhesion proteins, osteocytes respond to different stimuli in the context of exercise (19). Osteocytes express β1 and β3 subunits of integrins. Integrin β1 is related to α1 to α5 subunits, while integrin β3 only corresponds to αv. Using an Itga5 conditional gene knockout mouse model, it has been shown that integrin α5 plays an important role in regulating the anabolic response of bone to mechanical loading. Integrin α5 inhibits osteocytes apoptosis, increases endosteal osteoblasts and bone formation, and reduces osteoclasts and bone resorption. Moreover, integrin α5 increases load-induced Cx43 function and decreases the production and release of PGE2, which results in the upregulation of sclerostin and reduction of β-catenin. For bone loss, integrin α5 has the potential of becoming a new therapeutic target, especially in older individuals with reduced mechanical sensitivity (30).

#### 3.1.2 Connexins and gap junctions

In addition to integrins and focal adhesion, gap junctions and hemichannels, which are formed by connexins, play important roles in regulating cell-to-cell communication in the bone microenvironment (21). Six connexin molecules, either same or different types, compose a connexon, and two connexons form a gap junction on the surface of adjacent osteocytes. When connexons are only found on one side of a cell, they form hemichannels. Hemichannels and gap junctions are necessary in cell-to-cell messenger exchange (21). Gap junctions are most abundant in osteoblasts and osteocytes, and because connexin forms gap junctions, the expression of connexin family members, including Cx37, Cx40, Cx43, and Cx45, is regulated by the bone microenvironment (21). Under mechanical stimulus of exercise, Cx43 is expressed in osteocytes to orchestrate bone turnover and remodeling (21).

A study in mice with dominant-negative Cx43 mutants in osteocytes has demonstrated that osteocytic Cx43 is essential for anabolic function of bone in response to mechanical loading (31). Mechanical loading of tibial bone upregulated cortical bone mass and mechanical properties in control mice through increased PGE2, endosteal osteoblast activity, and reduced sclerostin. In contrast, in gap junction–impaired Δ130–136 mice, these bone anabolic responses were all impeded and endosteal osteoclast activity was enhanced (31).

#### 3.1.3 Purinergic receptors

Purinergic receptors also participate in the regulation of bone homeostasis by exercise because they can be activated by ATP as well as ADP, which are produced in various types of exercise. It has been reported that ATP is concentrated inside the cell, while extracellular ATP levels are only upregulated during cell lysis or in response to robust mechanical stimuli such as in high-intensity exercise (32). By using the MLO-Y4 osteocyte model, it has been demonstrated that osteocytes secrete ATP in response to mechanical stimulation of exercise in a dose-dependent manner (32).

Purinergic receptors were first classified into three types: G-protein–coupled receptors (GPCRs) adenosine receptors (P1), P2X ion channels, and P2Y G-protein–coupled receptors (22, 33). P2X and P2Y receptors have been detected in osteoblasts, osteoclasts, and osteocytes (20). In regulation of the bone environment in exercise, ATP is released, and P2 receptor and its downstream signaling are vital modulators. It has been reported that P2X7 receptor activation increases mineralization of osteoblasts (34). Furthermore, P2X7 knockout mice express bone mass reduction (34), and defective osteogenesis, which leads to wider calvarial sutures (35).

#### 3.1.4 Polycystins (PCs)

Polycystin-1 (PC-1) and polycystin-2 (PC-2) were first identified in renal primary cilia cells. By conjugating transiently, they form mechanosensitive receptor channel complexes, TRPP channels, and act as mechanosensitive channels. It is known that defects in PC-1 and PC-2 result in autosomal dominant polycystic kidney disease. However, the full spectrum of PC1 and PC2 effects has been difficult to establish (26, 27, 36–39).

Recently, it has been found that mice with the conditional deletion of PC1 in bone marrow mesenchymal stem cells (BMSCs), osteoblasts, and osteocytes display noteworthy decreases in bone mineral density as a result of the reduced osteoblast-related bone formation *in vivo* (37, 38). In the same way, the conditional deletion of PC2 in osteoblasts leads to a decrease in trabecular bone volume, bone mineral density, and cortical thickness *in vivo* (39). PC1, PC2, and TAZ react to the flow shear force, characterized in collaboration to each other, and accelerate osteoblast maturation by activating Runx2 and repressing PPARγ activities (40). Furthermore, human genome-wide association studies (GWAS) have suggested the relationship between PC2 and osteoporosis (41). At present, there is an agonist called triptolide, which can bind to PC2 and rescue Ca2+ signaling to diminish whole cyst formation in renal epithelial cells *in vitro* (42). Hence, triptolide seems promising in providing a new option for the treatment of osteoporosis, although additional experiments are needed to confirm its effects.

The effects of exercise on PC1/3 have rarely been investigated. Moderate-intensity exercise (20 m/min, 1 h) increased the quantity of PC1/3 in the pituitary gland (43), indicating that PC1/3 might be a possible regulator of bone response to exercise.

#### 3.1.5 Piezo1/2

Piezo proteins (Piezo1 and Piezo2), discovered in 2010, form mechanosensitive cation channels (44). Piezo channels are mechanosensitive ion channels located in the cell membrane and function as key cellular mechanotransducers for converting mechanical stimuli into electrochemical signals. Under mechanical stimuli, these channels are opened to allow cations to cross the membrane, which promotes cellular mechanotransduction to adapt to the microenvironment (45). Recently, some researchers have suggested that those Piezo proteins are important mechanosensitive channels in the bone microenvironment.

PIEZO1 and PIEZO2 show robust expression in primary articular chondrocytes. GsMTx4, a PIEZO-blocking peptide, reduces chondrocyte death by regulating the Ca2+ signaling pathway after mechanical injury, shedding light on cartilage injury therapy (28). Moreover, the Piezo channels are involved in bone disease and play pivotal roles in the transduction of mechanical forces in other bone cells (29, 46).

It has been reported that the Piezo1–YAP1–collagen pathway in osteoblasts is important for the regulation of bone mass *in vivo* and *in vitro* (47). Deficiency of Piezo1 in osteoblasts resulted in osteoporosis. In tail suspension assays, the hind limb of mice was unloaded for 8 weeks. The bone mass of these mice was reduced, supporting the hypothesis that PIEZO channels directly sense mechanical loading in skeletal cells. Piezo1 increased collagen II and collagen IX expression by promoting YAP nuclear translocation in osteoblasts. In contrast, in osteoclasts, Piezo1 knockout did not affect bone loss (47).

Li et al. verified the vital role of Piezo1 in sustaining bone homeostasis by regulating the sensitivity of osteocytes and osteoblasts to mechanical loading *in vivo* and *in vitro* (46). An *in vitro* study demonstrated that the activation of Piezo1 by Yoda1 mirrored the raise of Ca^2+^ influx outcomes of fluid shear stress on osteocytes. In line with the *in vitro* results, Yoda1 increased the bone mass of the mice (46).

#### 3.1.6 Wnt signaling and sclerostin 

**Figure 2:**
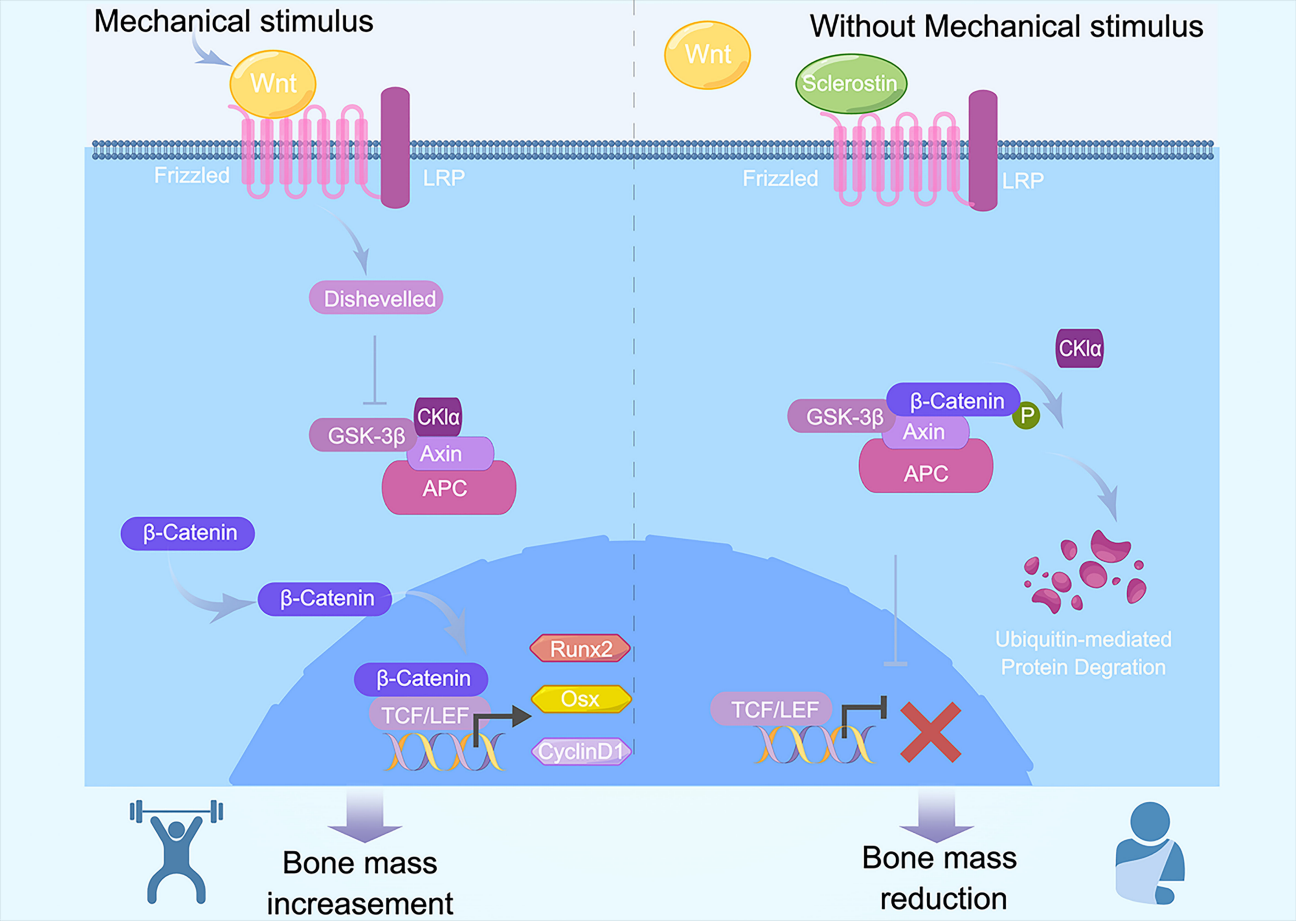
Canonical Wnt signaling and sclerostin in mechanical regulation of exercise. Exercises produce mechanical stimulus and increase Wnt1. Wnt1 binds with LRP5/6, subsequently activate intercellular Dvl, resulting in release of free β-catenin and translocation into nuclei. β-cateniin binds with TEF/LEF, contributes to induction and activation of Runx2, Osx, and cyclinD1. Osteoblast differentiation and bone mass increase is activated by Wnt and inhibited by the Wnt antagonists Frizzled and sclerostin. When without mechanical stimulus bring by exercise, Wnt1 decreased and β-catenin is ubiquitinated and degraded, inducing downregulation in the expression of downstream target gene, bone mass subsequently decreased.

Osteocytes sense mechanical loading transmitted to bone and downregulate the expression and secretion of sclerostin (25). Sclerostin competitively binds to the extracellular binding site LRP5/6, conveying an antagonistic effect on the Wnt/β-catenin pathway (48) (**Figure 2**).

The Wnt/β-catenin pathway is conservative in various organisms. It is important for cell differentiation, growth, apoptosis, and biological functions. It is also necessary for organism growth and development. So far, there are three types of Wnt signaling, including canonical Wnt signaling, non-canonical Wnt/Ca+ signaling, and Wnt/PCP signaling pathway (17). Canonical Wnt signaling is the most important regulator of differentiation from stem cells to osteoblasts and osteocytes.

Wnt signaling components, such as Wnt, β-catenin, GSK-3β, and Axin, play a pivotal role in bone metastasis regulation (17). Wnt1 binds with frizzled family member FZD and LDL receptor–related protein (LRP5/6). Subsequently, they activate intercellular Dvl (Dishevelled), thus forming a complex that destroys another complex that is composed with APC, Axin, GSK-3β, and β-catenin. As a result, released free β-catenin translocates into nuclei and binds with the transcriptional factor T cell factor/lymphoid enhancer factor (TCF/LEF). At last, expression of downstream target genes Runx2, Osx, and cyclinD1 increases. In contrast, without Wnt1, the β-catenin complex is ubiquitinated and degraded, inducing a decrease in the expression of downstream target gene (17). Wnt signaling activation results in bone mass increase (49).

A recent study has reported a new vibration-induced bone-enhancing protein, R-spondin 1 (50). As a Wnt pathway modulator, R-spondin 1 is secreted by young mesenchymal progenitor cells and promotes bone formation in osteoporosis in response to vibration (50).

It has been shown that the Wnt3a/β-catenin pathway in mice with type 2 diabetes mellitus is upregulated after downhill running. Subsequently, osteoblasts differentiation, osteogenic capacity, and bone morphological structure are enhanced. In a word, downhill running reverses osteoporosis by activating Wnt signaling in osteoblasts (51).

In another study, mechanical loading of the ulna decreased *Sost* transcriptional levels in wild-type mice, while HLU enhanced sclerostin expression (23). The phenomenon was in parallel with the increased level of sclerostin detected in human serum after prolonged bed rest and immobilization.

In summary, these findings suggest that mechanically responsive activation of receptors and iron channels on the surface of osteocytes, osteoblasts, and osteoclasts, ranging from integrins, connexin, purinergic receptors, Piezo1/2, and PCs to Wnt signaling pathway, is closely related to osteoporosis. In particular, Piezo1, PC1, and PC2 are innovative therapeutic targets for osteoporosis, and their activators may be able to treat osteoporosis. They may also be used to evaluate recovery after orthopedic surgery by detecting the levels of each target.

### 3.2 Indirect regulation by extracellular signaling molecules

Independent of the receptors on cell surface, exercise-induced regulation of bone environment may also occur through cytokines. Here, we summarize the common cytokines, such as inflammatory factors. Moreover, we address more promising myokines, such as irisin and myostatin.

#### 3.2.1 Cytokines

##### 3.2.1.1 Inflammatory factors

Cells sense and respond to mechanical strain fluctuations by adhesion structures such as integrins. Mechanical signals are then transferred into biochemical responses, regulating various processes from cytoskeleton change to cytokines secretion (52). This may lead to a disbalance between bone resorption and bone modeling (53).

The majority of proinflammatory cytokines such as IL-6, TNF-α, IL-1, and IFN-γ play a pivotal role in the treatment of osteoporosis through exercise (54–56). Exercise may improve inflammation by upregulating anti-inflammatory factors, which may have an effect on bone metabolism (57). It has been reported that high-intensity, low-impact, interphase exercise is an effective stimulus for producing proinflammatory cytokines such as IL-1, IL-6, and TNF-α, which shows a relationship between exercise, immunity, and the bone microenvironment (57).

Genetic variants of IL-6 are one of the risk factors for osteoporosis. It can increase the number of S1PR2 receptors on the surface of osteoclast precursors and accelerate the transformation from bone marrow-derived macrophage (BMDM) to mature osteoclasts (58). Zhou et al. reported that the beneficial effects of proper exercise on bone damage recovery were mediated through inflammation-inducing factors (59). The authors studied the osteoblast genesis mechanism in mice that continued exercising. At week 8, proinflammatory factors such as TNF-α and IL-6 were reduced through running or weight-bearing running, and the bone and bone marrow formation were promoted by weight-bearing exercise. Running or weight-bearing running may also be helpful for the recovery of patients with osteoporotic fractures. Zou et al. measured the levels of IL-1β, IL-6, and Cox-2 in serum and bone marrow; they found that ovariectomy led to a sharp increase in IL-1β in the bone marrow and IL-6 and Cox-2 in tibias, while exercise reduced the levels of IL-1, IL-6, and Cox-2 (60). That study suggested exercise as a robust method for the improvement of osteoporosis in postmenopausal osteoporosis rat models through inhibiting bone resorption and promoting bone formation, particularly in trabecular bone (60). Similarly, another study reported that after 6 weeks of moderate exercise training with estradiol therapy of ovariectomized (OVX) rats, serum analysis showed a significant increase in bone resorption markers and bone formation markers, from osteocalcin (OCN) and bone-specific alkaline phosphatase (ALP) to proinflammatory cytokines such as TNF-α and IL-6 (55). Hence, moderate exercise training improved osteoporotic alterations in OVX rats by inflammatory change and showed a potential to be an effective therapeutic strategy for postmenopausal osteoporosis (55). Mert et al. found a relationship between IL-6 levels and hip fracture in the study group after surgery, but TNF-α and IL-6 levels were not related to the occurrence of death and walking capability after the surgery. By measuring the levels of TNF-α and IL-6, they analyzed the relationship between inflammatory markers and postoperative mortality as well as walking capability. However, further clinical and functional confirmation is needed.

IFN-γ belongs to type-II interferons. The last developmental phases of osteoclasts can fuse to multinucleated osteoclasts through IFN-γ activation. IFN-γ induces the expression of NFATc1 and then the transcription of c-FOS (61). RANKL is a well-known messenger that starts the differentiation of osteoclasts. By increasing the quantity of MHC-II, IFN-γ activated T cells, and then the T cells secreted more RANKL (61). Inflammatory bone loss in the OVX mice associated with IFN-γ was rescued by wheel-running exercise. After that, CD8 T cells were activated and then secreted IFN-γ, which induced the NF-κB/MAPK pathway activation and inhibited osteoclastogenesis (56). After HA/β-TCP biomaterials were implanted into the mice, IFN-γ levels increased, which helped to treat osteoporotic fractures (59).

However, another study showed that running inhibited bone metabolism and levels of proinflammatory factors in male aged rats (62). In older rats on running training, the levels of TNF-α and IFN-γ from CD8(+) T cells were upregulated, and the number and activity of osteoblasts from the running rats were decreased (62). Inflammatory factors, such as TNF-α, IL-6, and IFN-γ, secreted by different cells in bone microenvironment. Their activities and effects depend on environmental heterogeneity. As a result, more robust studies are needed for this topic and further work will be necessary to allow for more complex and powerful experimental designs. Hence, it seems that we need more delicate settings to investigate the role of exercise in fighting osteoporosis by inflammatory regulation.

In addition to inflammatory cytokines, other factors associated with bone microenvironment, such as osteoprotegerin (OPG), can respond to mechanical stimuli. OPG is a decoy receptor for RANKL, and also influences bone remodeling. A prior study showed that moderate intensity endurance and vibration exercise upregulated OPG expression and downregulated the level of RANKL, thereby delaying osteoclast maturation and activity (63).

Overall, these findings indicate that cytokines, especially inflammatory factors, eventually modify the microenvironment in response to exercise or external mechanical stimuli, which changes cellular functions, influences bone homeostasis, and facilitates bone remodeling.

#### 3.2.2 Myokines

Exercise promotes the activity of different types of muscle, especially skeletal muscle and cardiac muscle. Thus, muscles can be considered organs that can release signal factors. These factors, such as irisin, cathepsin B, and lactic acid, are important messengers in the muscle–organ crosstalk. Recently, it has been shown that in response to exercise, many myokines have effects on mitochondrial metabolism and are beneficial for cognitive behavioral therapy (64). Moreover, some myokines such as myostatin (MSTN) and irisin could work in the bone environment and have an effect on bone remodeling. Their role in the muscle–bone crosstalk is essential for osteoporosis regulation under various types of exercise.

##### 3.2.2.1 Irisin

Irisin is a myokine that is a transcript of FNDC5 (65). The level of irisin expression is upregulated when people tremble in cold environment, which promotes brown adipocytes to burn energy and produce heat, as mitochondria are abundant in brown adipocytes (65).

Irisin not only allows for the crosstalk with adipose tissue, but also with bone, which are closely related to muscle in time and space. It has been shown in humans that after exercise, the levels of irisin produced from skeletal muscle and released to serum are increased (65, 66). Compared with aerobic exercise, endurance exercise can stimulate skeletal muscle to produce more irisin. Given that endurance exercise resembles trembling, the incretion of irisin is upregulated in response to both resistance exercise and trembling in cold conditions. However, in another study, after 8 weeks of training, the resistance exercise group and irisin injection group were more likely to have an increase in bone strength compared with the control group, but not compared with the endurance exercise group (67). This showed that resistance exercise can prevent bone mass loss and osteoporosis, although the relationship of endurance exercise and irisin secretion needs more investigation.

Under simulated microgravity, differentiation of osteoblasts can be increased by irisin (68). Meanwhile, irisin promotes osteoblastogenesis in the bony callus and accelerates the repair and growth of bone tissue after osteoporosis-related fracture. It is clear that irisin has a positive effect on osteoblasts. However, the effects and action mechanisms of irisin on osteoclasts are still unclear. Hu et al. found that irisin both rescued Ti-particle–impaired osteogenesis and mitigated the increase of wear-particle–induced RANKL from osteoblasts, thereby reducing bone resorption (69). In addition, irisin may lead to the inhibition of osteoclastogenesis by activating reactive oxygen species (ROS) signaling (69). This study revealed the mechanism of irisin in maintaining bone mass when binding to wear particles (69). But in another study, irisin activated downstream signaling pathways in cementoblasts by binding to the receptor integrin αV, and then to cells secreted RANKL and IL-6, which communicated with osteoclasts and promoted bone resorption (70).

Thus, it may be a novel pharmacologic modulator for the therapy of fracture and related complications of disuse osteoporosis. Moreover, moderate-intensity treadmill exercise promotes the circulation of irisin, so it is not difficult to deduce that after fracture, moderate-intensity treadmill exercise as soon as the body conditions allow may stimulate bone healing and recovery (71).

##### 3.2.2.2 Myostatin

Myostatin (MSTN), also known as growth differentiation factor 8 (GCD-8), belongs to the transforming growth factor-β family. It is also a negative regulator of muscle growth.

MSTN is secreted as a precursor protein; then, an N-terminal propeptide and a C-terminal growth factor dimer are generated, and an inactive latent complex is subsequently formed. Under the regulation by bone morphogenetic protein 1/tolloid proteases, the complex liberates mature myostatin (72, 73).

Organisms with MSTN knockout show BMD upregulation. A recent study in ovariectomized rats has demonstrated that after 10-weeks weight-bearing training, MSTN circulation increased, and bone loss was attenuated (74). In that study, the rats were ovariectomized and exposed to weight-bearing training. Specifically, the rats had to bear 35% of the body weight and run at 20 m/min, for 6 days/week in total. After 10 weeks, the BMD of the rats’ total femur and trabecular bone increased, compared with the control group; mechanically, Wnt1 and β-catenin mRNA and protein levels were upregulated (74).

##### 3.2.2.3 Others

In addition to MSTN and irisin, other myokines such as kynurenic acid (Kyna) also show important regulatory function in bone metabolism in osteoporosis (75). In the serum of ovariectomized mice, the concentrations of serum Kyna and kynurenine aminotransferases (Kats) were lower than in the control group. After treadmill-running exercise, the muscle levels of KATs and the serum concentration of Kyna increased. Meanwhile, the bone loss was reversed due to inhibiting osteoclast maturation through the Kyna/Gpr35/NFκB p65/Nfatc1 pathway and enhancing osteoblast proliferation (75).

Postmenopausal osteoporosis due to decreased estrogen levels and ovariectomized models show the characteristics of reduced estrogen levels (76). A recent study has demonstrated that estrogen signaling regulates expression and secretion of myokines that modulate osteoclast differentiation and activity. The authors used conditioned media that were collected from ovariectomized and skeletal muscle deficient in estrogen receptor α expression mice, with cytokine array, and found that estrogen deficiency altered myokines levels (76).

In summary, myokines, especially irisin and myostatin, can protect bone mass by acting on osteoblasts and osteoclasts, and by regulating the crosstalk in the bone microenvironment.

## 4 Types of osteoporosis and effects of exercise

A patient with osteoporosis has a low bone mass and deteriorated osseous microarchitecture, which results in weakened bone strength and an increased risk of fragility fractures. The Ministry of Health in the UK estimated 137 million women and 21 million men have a high osteoporotic fracture risk globally, and its prevalence is expected to double in the coming 40 years (1). Exercise is important in preventing and treating osteoporotic fractures. In older adults with higher levels of physical exercise, the incidence rate of hip fractures is 30%–50% lower than that in age-matched individuals with less exercise (77). In a prospective Epidemiology of Osteoporosis (EPIDOS) study, after following 6901 White women (≥75 years old) for 3.6 years, it was shown that low-intensity physical exercise increased the risk for proximal humerus fractures by more than two-fold (77).

Choice of exercise types requires careful consideration of risks and benefits of each manner of activity, as well as modern health status and physical fitness intensity. Physical exercise programs appear to have varying impacts on osteoporosis depending on their frequency, duration, and intensity (78). For patients with depression and osteoporosis, resistance training is a more universal and more reasonable choice than aerobic exercise, with evidence suggesting only benefits for depression (79). High-velocity, high-impact activity, such as jumping, is suitable for osteoporosis patients if they are able to tolerate the intensity. However, if patients present various clinical manifestations of osteoarthritis, it is better to avoid such activities. Moreover, considering that resistance training acts as a local stimulus to the bone microenvironment, to expand training into the whole-body skeletal strength, balance training should be encouraged to prevent falls (2).

Similarly, the most recent national guidelines from UK also recommend individuals with osteoporosis to undergo resistance and effective exercise programs to increase bone strength. Moreover, by taking part in physical activities, the risk of falls is reduced with the improvement of bone strength and balance. Vertebral fractures and falls caused pain. To reduce the pain, we should improve posture by undertaking spinal extension exercise (80).

### 4.1 Primary osteoporosis

Primary osteoporosis is most common in older population and postmenopausal women. There are two main forms of primary osteoporosis, including postmenopausal osteoporosis (type 1) and senile osteoporosis (type 2) (81).

#### 4.1.1 Postmenopausal osteoporosis

Due to reduced estrogen levels in postmenopausal women, osteoporosis and osteoporotic fractures are common (82). It has been reported that 80% of individuals with osteoporosis are postmenopausal women (83), and approximately half of all postmenopausal women sustain a fracture due to osteoporosis (84). Early studies have shown that estrogen and mechanical stimulation corporately inhibit osteoclast (OC) generation (85), suggesting that they may work better against postmenopausal osteoporosis.

Recent research has demonstrated that effective exercises as indicated intervention strategies can prevent and control postmenopausal osteoporosis (86). By measuring and comparing the BMD of postmenopausal athletes with different levels of physical activity, Yang et al. concluded that long-term exercise effectively prevented bone loss in female athletes and improved their musculoskeletal fitness (87). In addition, wheel-running exercise prevented bone loss in OVX mice (56). RNA-sequencing technology in the peripheral blood mononuclear cells (PBMCs) of OVX rats was also used to investigate the effect of moderate-intensity treadmill exercise on the sensitive genes related to bone mass (88). It was found that moderate-intensity treadmill exercise improved bone mass by increasing CCL2 and other genes in PBMCs. Thus, PBMCs can be a useful tool in the peripheral blood for supervising the exercise effect on bone strength in postmenopausal osteoporosis (88).

Although exercise is regarded as an effective method to deter bone loss triggered by estrogen deficiency in postmenopausal women, the optimal training system for peak bone growth has not yet been outlined. A previous study has found that interval training is not better than endurance training regarding OVX mice bone growth (89). However, since the authors did not give a clear regime to support the training methods (89), further experiments investigating the duration and frequency of the training schedule are needed.

#### 4.1.2 Senile osteoporosis

Age-related bone loss is associated with accelerated bone resorption and lower bone formation, resulting in osteoporosis and related fractures. Resembling other types of osteoporosis conditions, age-related bone loss is associated with a systemic bone loss related to trabecular and cortical compartments, osteopenia, osteoporosis, and subsequently high fracture risk (90). The number of patients who suffer from age-related bone fracture has substantially increased because of the global aging. Long-term care after fracture has also increased, which dramatically adds to the economic and societal burden. The effects of osteoporotic fractures on the health of older adults are also cataclysmic.

Due to widespread aging-induced changes in cellular function, tissues and organs change phenotypically, including those in the bone microenvironment. In elderly people, the production of mesenchymal stem cells (MSCs) and hematopoietic stem cells (HSC) is exhausted, and the regulation of cellular signaling is changed, resulting in the upregulation of pro-adipogenic, pro-osteoclastogenic, and anti-osteogenic factors. These are all systemic and bone-localized cytokines that affect the cellular crosstalk in the bone microenvironment (91). Impaired autophagy (92), excess ROS (93), DNA damage (94) and cellular senescence (95) may be potential therapeutic targets in treating osteoporosis.

Physical activity is considered an effective support for improving bone health in older people (90). The number of HSCs is upregulated by physical activity or exercise (96, 97). Exercise training increases the survival rate of bone marrow transplantation mice from 25% to 82% (98). In aged OVX mice, resistance exercise conducted by voluntary tower climbing reduces the number of osteoclasts compared to the control groups (99). It has also been reported that vibrations of the ankle muscles at a low intensity improve balance in elderly people at a high risk of falls (100), which may help prevent age-related osteoporosis complications.

In conclusion, the beneficial effects of exercise against aging-related osteoporosis have been reported in many studies. However, there are still many unsolved issues, such as improvement of its clinical efficacy and reduction of time spent on exercise by elderly patients.

### 4.2 Secondary osteoporosis

Osteoporosis can occur secondary to other traumatic conditions and metabolic diseases, such as long-term and large-dose drug intake (101, 102), hyperparathyroidism (103), and rheumatoid arthritis (104). Here, we address the two most common types of secondary osteoporosis.

#### 4.2.1 Disuse osteoporosis

Immobilization and weightlessness have important effects on body physiology; because they involve a lack of mechanical loading, they lead to the reduction in skeletal integrity. As a result, disuse is one of the major risk factors for osteoporosis (105). Disuse osteoporosis has become a common clinical problem that impairs physical function. Many diseases contribute to disuse osteoporosis, such as multiple sclerosis (106) and neurological disorders (107); however, unloading is the least uncommon factor in skeletal deterioration. The affected limb of a patient who undergoes amputation tends to lose bone mineral density more quickly than the fit limb (108). It has been demonstrated in patients with spinal cord injury that bone loss reaches a peak after three months (L. 109, 110). The greatest bone mass reduction always occurs below the level of injury, but it also occurs throughout the body (L 109). Disuse conditions favor a combination of high bone resorption and low bone formation (111), leading to immediate bone loss and eventually to osteoporosis with increased falling and fracture risk.

Exercise increases osteoblast collagen expression and mechanical strain affects collagen arrangement, which have an effect on new bone formation, thereby strengthening the skeleton. Bed rest or weightlessness, without physical activity, negatively regulates bone by affecting the activities of osteoblasts and osteoclasts (112). It has been reported that fluid flow upregulates Runx2 expression by both the classical and nonclassical Wnt pathways in mouse BMSCs, and promotes BMSCs differentiation to osteoblasts, while mechanical unloading inhibits β-catenin expression and suppresses osteoblast proliferation (113).

In recent years, there has been an increased concern about mechanoresponsive sensitivity in human fracture recovery to help patients’ fast and robust return to normal life. Qian et al. found that, for bedridden patients, increased miR-138-5p levels in bone specimens correlated with a lower degree of bone formation (114). They found that under different mechanical conditions, miR-138-5p directly targeted MACF1 to inhibit osteoblast maturation (114). By bone-targeted inhibition of miR-138-5p, the bone anabolic response to mechanical loading became sensitized in miR-138-5p transgenic mice, and bone formation was accelerated. They identified miR-138-5p as a mechanoresponsive miRNA, and inhibition of miR-138-5p in osteoblasts may be a novel bone anabolic sensitization strategy for improving disuse osteoporosis (114).

Altogether, physical exercise is an effective therapeutic strategy to help patients with immobilization and improve their quality of life. Improvement of sensitivity to mechanical stimuli is a feasible approach for accelerating patients’ recovery.

#### 4.2.2 Glucocorticoid-related osteoporosis

Glucocorticoids are broadly used to suppress inflammation in many immune diseases, such as rheumatic diseases and those arising after organ transplantation (101). Glucocorticoid-related osteoporosis is the most critical complication induced by glucocorticoids (101).

Early studies have found that glucocorticoids regulate differentiated bone cell functions and osteoclastogenesis to regulate the balance between bone resorption and bone formation (115). Glucocorticoid-related osteoporosis is a predominant form of secondary osteoporosis (116). Its major cause is likely the impairment in bone formation (116). Normally, BMSCs can differentiate into osteogenic, adipogenic, and chondrogenic cells, which is known as tri-lineage differentiation potential (117). However, in glucocorticoid-related osteoporosis, the quantity and activity of osteoblasts decreases, while those of fat cells increases (118). However, because of other side effects and off-target effects, current drug treatments are a long way from achieving satisfactory effects (102).

Glucocorticoid overdose and unsatisfied treatments lead to detrimental side effects on bone as well as on muscle, cartilage, and fat mass. Healthy diet and regular physical exercise might mitigate these conditions (119). As for physical exercises, the BBC guidelines on postmenopausal osteoporosis recommend focusing on impact training, body balance, strength, and resistance training (120). It is also suggested that regular exercise is an effective therapeutic and preventive strategy with few side effects in glucocorticoid-related osteoporosis in adults (119).

In conclusion, exercise has a positive effect on the treatment of glucocorticoid-related osteoporosis, although more studies are needed to investigate the physiological and pathological mechanisms involved.

## 5 Conclusions and perspective

In summary, mechanically speaking, the exercise-related regulation of osteoporosis is complex and likely involves multiple pathways, including direct and indirect mechanisms. This includes mechanical signaling transduction from receptors on the cell surface, such as PC-1/2 and Piezo1/2, to cytokines in the bone microenvironment, ranging from inflammatory factors to myokines. The changes in biochemical markers of bone turnover and consequently the change in bone mass might be seen as a manifestation of direct or indirect exercise effects, but more studies are needed to further clarify fine regulation.

Considering the advantages of exercise in the regulation of osteoporosis, such as low costs, easy performance, and few adverse effects, exercise has a huge potential in the future in osteoporosis treatment and prevention. Hence, exercise has become increasingly appreciated by doctors and researchers (**Table 1**). Exercise effects in multiple classes of osteoporosis have also been well documented. Recent UK guidelines also provide dedicated recommendations (80). We need further studies to determine the mechanisms by which exercise regulates different types of osteoporosis, reduce the exercise sensitive threshold of patients, increase exercise regulation efficiency, and prevent harm from hypermobility. All of these aspects would lessen the pain and fracture risk of osteoporosis patients.

**Table 1:** Recommendation for exercise.

Species	Types	Mechanisms/Effects	References
*older rats*	*running training*	*levels of TNF-α and IFN-γ from CD8(+) T cells were upregulated; number and activity of osteoblasts decreased;*	(62)
*human*	*moderate-intensity endurance and vibration exercise*	*OPG expression was upregulated; level of RANKL was downregulated;*	(63)
*human*	*aerobic exercise and endurance exercise*	*serum irisin levels increased;*	(65, 66)
*human*	*resistance exercise*	*bone strength increased; bone mass loss was prevented;*	(67)
*human*	*moderate-intensity treadmill exercise*	*serum irisin levels increased;*	(121)
*human*	*microgravity stimulation*	*serum irisin levels increased; differentiation of osteoblasts increased;*	(68)
*human*	*resistance and effective exercise programs*	*bone strength increased*	(78)
*patients after osteoporosis-related vertebral fracture*	*spinal extension exercise*	*pain was reduced;*	(80)
*postmenopausal female athletes*	*long-term running*	*bone loss was prevented; musculoskeletal fitness was improved;*	(87)
*OVX mice*	*wheel-running exercise*	*bone loss was prevented;*	(56)
*OVX rats*	*moderate-intensity treadmill exercise*	*bone mass was improved by increasing CCL2;*	(88)
*OVX mice*	*interval training*	*bone growth was not affected;*	(89)
*OVX mice*	*endurance training*	*bone mass increased;*	(89)
*OVX mice*	*voluntary tower climbing*	*number of osteoclasts decreased;*	(99)
*elderly people at a high risk of falls*	*vibrations of the ankle muscles at a low intensity*	*body balance improved;*	(100)
*mice*	*vibration training*	*R-spondin 1 was more secreted; bone formation in osteoporosis was enhanced;*	(50)
*type 2 diabetes mellitus mice*	*downhill running*	*Wnt3a/β-catenin pathway was activated; osteoblasts differentiation ability was enhanced; bone mass increased; osteoporosis was reversed;*	(51)

## Author contributions

All authors were involved in conceptualizing the paper. XC led the drafting of the manuscript. SX and HZ reviewed, edited, and approved the final paper. All authors contributed to the article and approved the submitted version.
